# Supplementary material for: Efficient Cardiac Differentiation of Human Amniotic Fluid-Derived Stem Cells into Induced Pluripotent Stem Cells and Their Potential Immune Privilege
Source: Int J Mol Sci. 2020 Mar 29;21(7):2359. doi: 10.3390/ijms21072359 (PMC7177657; doi:10.3390/ijms21072359)
Supplement: Supplementary file 1 [file ijms-21-02359-s001.pdf]

Supplemental table S1. The culture medium and supplemental reagents used for cardiac differentiation.

Medium / chemical	Company	Catalog number
α-MEM medium	Gibco, Invitrogen	11900024
Fetal bovine serum	HyClone	SH30070.03
RPMI	Gibco, Invitrogen	11875119
B27 (minus insulin)	Gibco, Invitrogen	A18956-01
B27 (plus insulin)	Gibco, Invitrogen	17504-044
bFGF	PepproTech	AF-100-18B
CHIR 99021	Tocris	4423
IWR-1	Sigma	I0161

Supplemental table S2. The antibody used for flow cytometry, immunofluorescence (IF) and immunohistochemistry (IHC) staining.

antibody	Company	Catalog number
Nanog	BD Biosciences	560483
SSEA4	BD Biosciences	560128
OCT4	BD Biosciences	560329
HLA-ABC (for flow cytometry)	BD Biosciences	555553
HLA-ABC (for IF staining)	Abcam	ab70328
HLA-DR	BD Biosciences	555812
cTnT (for flow cytometry)	BD Biosciences	565618
cTnT (for IF staining)	Abcam	ab8295
α-actinin	Abcam	ab68194
Annexin V	BD Biosciences	640945
CD3	Abcam	ab5690
CD20	Abcam	ab64088
CD68	Abcam	ab125212
Connexin 43	Sigma	C6219
Goat anti mouse Alexa Fluor 488	Abcam	ab150117
Goat anti rabbit Alexa Fluor 568	Abcam	ab175471
Ki67	Cell Signaling Technology	11882s
